# Fusing Visual Attention CNN and Bag of Visual Words for Cross-Corpus Speech Emotion Recognition

**DOI:** 10.3390/s20195559

**Published:** 2020-09-28

**Authors:** Minji Seo, Myungho Kim

**Affiliations:** Department of Software Convergence, Soongsil University, 369, Sangdo-ro, Dongjak-gu, Seoul 06978, Korea; porito2@soongsil.ac.kr

**Keywords:** speech emotion recognition, cross-corpus, bag of visual words, visual attention, convolutional neural network, log-mel spectrograms

## Abstract

Speech emotion recognition (SER) classifies emotions using low-level features or a spectrogram of an utterance. When SER methods are trained and tested using different datasets, they have shown performance reduction. Cross-corpus SER research identifies speech emotion using different corpora and languages. Recent cross-corpus SER research has been conducted to improve generalization. To improve the cross-corpus SER performance, we pretrained the log-mel spectrograms of the source dataset using our designed visual attention convolutional neural network (VACNN), which has a 2D CNN base model with channel- and spatial-wise visual attention modules. To train the target dataset, we extracted the feature vector using a bag of visual words (BOVW) to assist the fine-tuned model. Because visual words represent local features in the image, the BOVW helps VACNN to learn global and local features in the log-mel spectrogram by constructing a frequency histogram of visual words. The proposed method shows an overall accuracy of 83.33%, 86.92%, and 75.00% in the Ryerson Audio-Visual Database of Emotional Speech and Song (RAVDESS), the Berlin Database of Emotional Speech (EmoDB), and Surrey Audio-Visual Expressed Emotion (SAVEE), respectively. Experimental results on RAVDESS, EmoDB, SAVEE demonstrate improvements of 7.73%, 15.12%, and 2.34% compared to existing state-of-the-art cross-corpus SER approaches.

## 1. Introduction

Emotion recognition (ER) plays an important role in human-computer interaction (HCI) [1]. During the last few years, numerous approaches have been proposed using different modalities (e.g., speech, facial expressions, and gestures) [2,3,4,5]. Speech is a useful modality in HCI research because of its different strengths, tremors, and speech rates depending on the emotional state. Therefore, speech emotion recognition (SER) is a useful research area in many ER challenges in terms of communicating human emotions and for computers using acoustic features.

There are two key steps in SER: (i) Extracting the appropriate acoustic features from speech signals in utterances and (ii) identifying the emotional state in the speech signal. There are four primary types of acoustic features that can be extracted from speech signals [6]: (i) Continuous features such as pitch and energy; (ii) features related to aspects of voice quality (e.g., harsh, tense, and breathy); (iii) spectral features, such as linear predictive coefficients, mel-frequency cepstral coefficient (MFCC), and log-frequency power coefficient; and (iv) Teager Energy Operator (TEO)-based features, such as the normalized TEO autocorrelation envelope area. Traditional SER studies [7,8,9,10,11] classify emotions using low-level features based on the hidden Markov model (HMM), Gaussian mixture model (GMM), support vector machine (SVM), and numerous other classifiers. In recent years, deep-learning models (deep neural networks, convolutional neural networks (CNNs), and long short-term memory (LSTM)) have emerged in several domains, such as computer vision, speech recognition, and natural language processing (NLP). Because deep-learning models can handle more complex features, recent SER studies have used deep-learning models to identify speakers’ emotions using high-level features from utterances. Many SER systems learn spectral features of utterances as high-level features, such as gammatone, short-time Fourier transform (STFT), and mel-spectrogram, which are obtained as 2D visual data by converting the time-based signal into the frequency domain utilizing the Fourier transform and deep-learning models such as CNN and LSTM [12,13,14,15].

The speech dataset has different acoustic features depending on the culture, context, and language of the speakers. Therefore, the SER classification performance varies with speech datasets. However, due to a lack of variety within speech datasets, it is difficult to classify the emotional state of speakers in a new speech dataset that has not been learned. Therefore, recent studies have considered the cross-corpus SER to classify emotions on a target speech dataset using a pretrained model on other speech datasets. Numerous researchers have attempted to achieve improved cross-corpora accuracy. Accordingly, transfer learning has primarily been adopted for cross-corpora.

Transfer learning [16] is a machine-learning technique that aims to improve the performance of a target domain with a small dataset by using knowledge from previously learned tasks with large datasets. The underlying concept is to not only leverage the pretrained model’s weighted layer, but also to selectively retrain some of the pretrained layers. In other words, some initial layers are frozen to capture generic features of the pretrained domain, and the remaining layers are fine-tuned to learn more specific features of the new domain. When the source dataset has a larger number of training samples than the target dataset, the method of transferring and fine-tuning layers is effective in assisting the learning target dataset. Speech datasets are composed of emotion-specific utterances with different language, context, and recording times. Therefore, transfer learning is an effective method for SER research when training small datasets utilizing the trained weights of large datasets.

To achieve better performance in the speech dataset regardless of the utterance type, we propose a method for identifying emotions by leveraging the previously trained weights in a large dataset. In the proposed method, we use a log-mel spectrogram, which adopts mel scaling, which computes a logarithmic scaling of the repetition frequency to represent human auditory characteristics representing the 2D spectrogram to a spectrogram where the horizontal axis is the time, and the vertical axis is the frequency of the speech signal and log-amplitude scaling to provide flexible and robust features. We classify emotions using the log-mel spectrogram by learning the high-level neural features of a spectrogram with a 2D CNN model and visual attention techniques. In our proposed visual attention CNN (VACNN) model, by emphasizing the target-specific channels in the channel-wise aspect and the most informative region of feature map in the spatial aspects through the visual attention technique [17], the CNN model obtains more useful high-level information in the spectrogram for improved emotion-classification performance.

Our VACNN model is pretrained in a large dataset and is applied to fine-tune small datasets for emotion classification. In addition, when learning small datasets, we utilize the bag of visual words (BOVW) method [18] that represents each image by a histogram of the visual words to find local and global features on the log-mel spectrogram to support the fine-tuned VACNN model. In computer vision, feature descriptors (e.g., scale-invariant feature transform (SIFT), histogram of oriented gradients (HOG), and speeded-up robust features (SURF)) are primarily used to detect the edge and texture information of an image. The BOVW adapts the bag of words (BOW) method used in NLP and implemented image features on the feature descriptor as the “words” of BOW. The visual words represent local features in the image, and the BOVW creates a visual vocabulary by grouping visual words based on their similarities. Because the BOVW represents a feature vector by creating a sparse histogram, the BOVW is useful in capturing the local and global features in log-mel spectrograms. Additionally, local and global features are expressed as attention weight through the attention mechanism, so that the features learned by the convolution layer can be used to obtain local and global information related to speech emotion in corpora. Moreover, methods that train spectral features using CNN for the source speech dataset and retraining for the target speech dataset have never been used before in the SER task. This leads to reduced training times and enables improved CNN model performance.

This study provides two key contributions:

(1) As a pretraining model, we train a log-mel spectrogram with a 2D CNN model with visual attention modules. Visual attention consists of two modules: (1) A channel-wise attention module that enhances convolutional features by the relationship between channels, so the model is able to find informative features for feature refinement, and (2) a spatial attention module that assigns weights to each pixel on convolutional features by concentrating spatial locations. Thus, the more target-specific features are learned to improve the classification performance.
(2) We designed the VACNN model for pretraining a large speech dataset and fine-tuning a small speech dataset to identify the emotion in utterances. Through fine-tuning, the proposed model can consider the generic features of the pretrained layer and the specific features obtained by retraining the pretrained layer. In addition, the textual information extracted from the log-mel spectrogram through the feature descriptor is represented as a global statistic feature and utilized as context attention weight in our VACNN to improve performance for SER and cross-corpus SER. Our method exhibits a better overall accuracy of 7.73%, 15.12%, and 17.80% for Ryerson Audio-Visual Database of Emotional Speech and Song (RAVDESS) [19], the Berlin Database of Emotional Speech (EmoDB) [20], and Surrey Audio-Visual Expressed Emotion (SAVEE) [21], respectively.

The remainder of the paper is organized as follows: In Section 2, we review existing SER literature. In Section 3, we describe the steps of our proposed method. In Section 4, we describe our experimental results and compare them with the state-of-the-art in the literature. Finally, in Section 5, conclusions and future work are discussed.

## 2. Related Works

The SER field of research classifies the emotional state of a speaker by analyzing the digital signal extracted from their utterances. In recent years, many researchers have conducted various studies to classify emotions by analyzing various types of acoustic features extracted from speech signals. Commonly, SER research uses a two-way method to analyze low-level features and utterance-based spectral features.

Studies that use low-level features classify emotions by analyzing acoustic feature spaces, primarily using SVM, GMM, HMM, and K-nearest neighbor (KNN). Sun et al. [9] applied decision tree SVM to low-level features selected by the Fisher feature selection method in utterances. Then, they achieved 83.75% and 86.86% accuracy for the Chinese Emotional Speech Corpus (CASIA) [22] and the EmoDB database, respectively. Kuchibhotla et al. [23] classified emotions through linear discriminant analysis, regularized discriminant analysis (RDA), SVM, and KNN using low-level features selected using feature subset selection techniques viz., sequential forward selection, and sequential floating forward selection (SFFS). They achieved 92.6% accuracy for six emotions on EmoDB and 90.5% accuracy for six emotions in the Spanish emotional speech database when they applied RDA to selected features using the SFFS method. Noroozi et al. [24] selected 88 low-level features, including pitch, intensity, MFCC, zero-crossing rate (ZCR), and filter bank energy parameters in utterances. Then, they used SVM or random forest, with and without applying principal component analysis (PCA) to classify emotions in utterances. They reported weighted accuracies of 56.07%, 65.28%, and 47.11% for SAVEE, Ryerson Multimedia Laboratory (RML) [25], and eNTERFACE [26] datasets using Random Forest, respectively. Fahad et al. [27] described a method to represent the discriminative features from MFCC through a GMM and recognized emotions in utterances with HMM. They reported a weighted accuracy of 69.5% for the interactive emotional dyadic motion capture (IEMOCAP) databases [28]. In the studies discussed above, they primarily learned low-level features using machine learning, and considered only general acoustic features. This has the disadvantage of ignoring the relationship between features that can be obtained from representations of utterances.

Other studies using spectral features, such as STFT and log-mel spectrograms, classify emotions using deep-learning models. Misramadi et al. [29] combined bidirectional LSTM (BiLSTM) with an attention mechanism to learn both the frame-wise raw spectral features and low-level features. They attached a weighted pooling layer to the attention to compute the score for the contribution of frames. They achieved weighted accuracy of 61.8% and 63.5% for IEMOCAP. Tao and Liu [30] proposed a new variation of LSTM, advanced long short-term memory (A-LSTM), which applied a weighted pooling Recurrent Neural Network (RNN) scheme. It could leverage the advantage of flexible time dependency modeling capability. They applied A-LSTM to low-level features and sequential acoustic features of IEMOCAP for SER and achieved an accuracy of 58.7%. Because they primarily learn contextual information of sequence acoustic features using LSTM-based models, they could ignore local correlations on the 2D spectral features depending on emotions.

Tarantino et al. [31] used the global windowing method on top of the already extracted frames to express relationships between datapoints, and applied self-attention to extract 384 low-level features to weight each frame based on correlations with the other frames. Then, they classified emotions using a CNN model and achieved a weighted accuracy of 64.33% for IEMOCAP. In this work because they applied windowing to an existing frame to overlap frames, frame related emotions could not receive a weight score depending on which frame is overlapped by the global window.

Hararolasvadi and Demirel [32] selected the most discriminant frames using K-means clustering for low-level features, including MFCC, pitch, intensity, and filter-bank energies. Later, for several frames, they learned the spectra-temporal features from STFT through a 3D CNN model. They reported weighted accuracies of 81.05%, 77.00%, and 72.33% for SAVEE for the six emotions, RML, and eNTERFACE’05 datasets, respectively. In this research, because the 3D CNN model learned only selected *k* frames, the CNN model could ignore local correlations between spectral features on unselected frame related emotions.

Mustaqeem and Kwon [33] revealed the amount of energy transmitted by a sound wave is correlated to the amplitude of a sound wave. The amplitude of a sound wave denotes the maximum displacement of an element in the middle from its rest location. They computed the maximum amplitude in each frame using the peak value of sinusoidal wave function. The adaptive threshold method [34] was used to remove the noises and silent portions, and, finally, a new audio file with the same sample rates without any noise and silent signals was reconstructed. With this preprocessed audio, they extracted STFT and used CNN and FC layers for SER. They achieved accuracies of 81.75% and 79.5% for IEMOCAP and RAVDESS, respectively. This method has a drawback in that the classifying emotions can be time consuming because the audio file must be analyzed and converted to audio without noise or silence for preprocessing. In the aforementioned studies [29,30,31,32,33], local correlations between spectral features could be ignored by using normalized spectral features from pre-processing.

In recent years, cross-corpus SER studies have been performed to classify emotions for different languages or genders. Schuller et al. [35] initially investigated cross-corpus SER studies using various low-level features through an SVM. Zong et al. [36] designed a domain-adaptive least-squares regression (DaLSR), which jointly trained the least-squares regression (LSR) model with the labeled training samples from the source speech corpus. They achieved the best weighted accuracies of 52.27%, 36.40%, and 30.19% in EmoDB using eNTERFACE, eNTERFACE using EmoDB, and AFEW 4.0 using EmoDB, respectively. Huang et al. [37] used a two-stage PCANet to cross-corpus SER. They train feature extractors for source data, unlabeled target data, and generated intermediate data by PCANet with filter alignment and fed output of three feature extractors as the final features into the source data. They trained the source data with SVM and tested the target data. They achieved unweighted accuracies of 61.41% and 63.75% for EmoDB using Airplane Behavior Corpus (ABC) [38], and ABC using EmoDB, respectively. In these studies, they adopted unsupervised learning, so it could learn irrelevant representations because of the absence of the label information.

Zhang et al. [39] designed transfer sparse discriminant subspace learning, which combines discriminative subspace learning, graph-based distance metric, and feature selection. They achieved an accuracy of 49.26%, 42.44%, and 42.42% for EmoDB using BAUM-1a, eNTERFACE using EmoDB, and BAUM-1a [40] using EmoDB, respectively. Latif et al. [41] measured cross-corpus SER performance using a low-level feature. They applied a deep belief network with three restricted Boltzmann machine layers or sparse autoencoder and SVM to the source domain dataset and then applied transfer learning for the target domain dataset. For positive or negative emotions, they learned acoustic features on FAU Aibo [42] emotion corpus and IEMOCAP and then transferred these pretrained features when training the EmoDB, SAVEE, and EMOVO corpus [43]. This research used captured feature distribution, so it ignores the specific features of deep learning methods in which features of the source dataset are not transferred to the training of the target dataset.

Liu et al. [44] designed a deep domain-adaptive convolutional neural network model, which combined the CNN and domain-adaptive layer based on the maximum mean discrepancy (MMD) regularization method. They learned salient features from the speech spectrograms and reduced the distribution mismatch between the source and target datasets. They achieved an unweighted accuracy of 49.93% and weighted accuracy of 58.13% for EmoDB using eNTERFACE, unweighted accuracy 38.10%, and weighted accuracy 38.10% for CASIA using EmoDB, and unweighted accuracy of 31.59% and weighted accuracy of 31.68% for eNTERFACE using CASIA. This method uses MMD methods, which can ignore class weight bias, so MMD can be minimized by either learning domain-invariant representation or preserving the class weights in the source domain. In existing cross-corpus studies, the discrepancy between feature distributions of different corpora have been considered. This can lead to performance reduction because it ignores the target-specific features of spectral features. In order to achieve better performance, we fused the fine-tuned our VACNN model pretrained on the source dataset and the feature vector with the BOVW. It considers target-specific features of spectral features and improves performance of cross-corpus SER. The proposed architecture and methodology in our work are described in detail in next section.

## 3. Proposed Methodology

In this study, to identify the emotions, we describe a model that learns the log-mel spectrogram on a large dataset, and then propose a method that fine-tunes the pretrained model when learning a small dataset. We use the characteristics of the log-mel spectrogram to recognize emotions in utterances.

**Figure 1:**
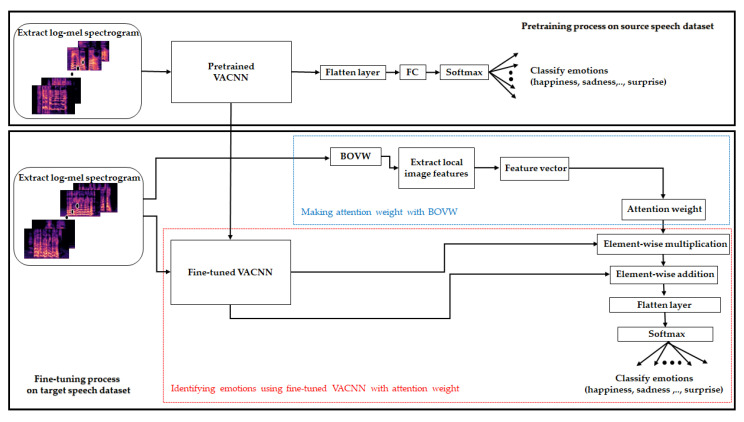
Overall architecture of our pretraining and fine-tuning models for SER.

Our method consists of two steps, as illustrated in Figure 1. First, we designed and pretrained our VACNN model to a log-mel spectrogram extracted from the utterance of the large dataset (Figure 1, top). The VACNN model is designed as a 2D CNN model that includes convolution blocks and a spatial- and channel-wise visual attention module. The convolution block is composed of convolution layers, group normalization (GN) [45], and rectified linear unit (ReLU) to learn high-level neural features. In addition, both visual attention modules assist VACNN to capture the refined features in the spatial- and channel-wise aspects. Second, we fused the fine-tuned VACNN model and the attention weight of visual vocabulary for the log-mel spectrogram to identify the emotions for a small dataset (Figure 1, bottom). We fine-tuned with the pretrained VACNN model on a large dataset in the first step. To improve the fine-tuned VACNN, we extracted feature vectors from local image features on a log-mel spectrogram using the BOVW and expressed it as an attention vector to assist VACNN. For joint learning with feature vectors of BOVW, we computed the element-wise multiplication of the attention vector and the fine-tuned VACNN. Then, we computed the element-wise sum to summarize the features of high-level neural features in the VACNN and the feature vector with the BOVW. Finally, a fully connected layer and softmax classifier were used to classify emotion in the log-mel spectrogram on a small dataset. In the second step, we captured general and specific features using the fine-tuned model (red block in the fine-tuning process), and utilized global static features represented by visual vocabulary using the BOVW to capture the informational features in the target dataset (blue block in the fine-tuning process).

### 3.1. Visual Attention Convolutional Neural Network for Pretraining

**Figure 2:**
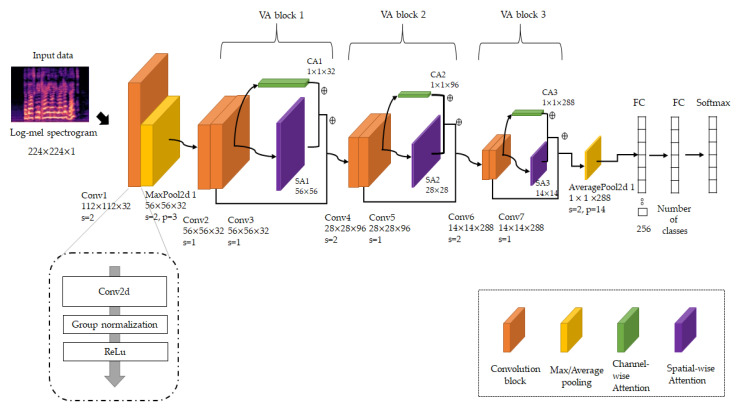
Architecture of the proposed pretrain VACNN model.

We designed a 2D CNN model to capture high-level neural features of the log-mel spectrogram on the source dataset for SER. We used the log-mel spectrogram as a grayscale image shape with 224 ×224×1 as the image height and width size, and the number of channels, where each pixel has a value from 0 to 255 for black and white shading. To identify emotions in utterances, our model designs similar architecture to residual neural network (ResNet) [46], which is mainly used as a backbone model for vision recognition and designs channel and spatial-wise attention module inspired by convolutional block attention module (CBAM). The proposed VACNN model for the source speech dataset is shown in Figure 2.

We first obtained 112 × 112 feature maps, which have high-level features extracted from 3 × 3 convolution layers (stride = 2), which have 32 filters. To obtain the fine-grained features, we also applied GN and ReLU after the convolution layer. We identified the three processes (convolution-GN-ReLU) as a convolution block. The GN is inspired by layer normalization that normalizes along the channel dimension, and instance normalization that normalizes each sample. To utilize the advantage that the computations of layer and instance normalization are independent of batch size, GN divides the channels into groups and computes normalization. We denoted the number of groups G and the number of channels C. After the convolution layer, GN decides the number of channels per group as C/G. For C/G, GN uses a feature-normalization method, such as batch normalization along the height and width axes. Because GN’s computation is dependent on batch size, GN can show stable performance in a wide range of batch sizes. Therefore, we used GN to normalize the feature maps with high-level features on a log-mel spectrogram, such that the model can be normalized stably, regardless of the small batch size according to the number of training samples. In addition, we applied the ReLU function to activate the model. Then, we downsampled the content of feature maps to 56 × 56 by using 3 × 3 max pooling (stride = 2) to reduce the execution time while maintaining their salient features.

Next, we designed three VA-blocks to sequentially learn high-level neural features. Each VA block has two convolution blocks as a first step. The convolution block is composed of 3 × 3 convolution layers, GN, and ReLU activation, but shows a difference in stride size. In the first VA block, both convolution blocks use a 1 × 1 stride with a convolution layer, which is convolved with the input at every pixel with overlapping receptive fields, resulting in a 56 × 56 × 32 refined feature map. Otherwise, the second uses a 2 × 2 stride with convolution layers in the first convolution block to capture high-level neural features with a downsampled feature map of 28 × 28 × 96, using a 1 × 1 stride with convolution layers in the second convolution block to find cross-channel correlations. The third VA block uses two convolution blocks with the same structure as the second VA block with 288 filters and obtains 14 × 14 × 288 refined feature maps. Each convolution block in the VA blocks has a normalized layer by GN and is activated by ReLU. In this model, we used 3 × 3 filters with convolution layers to learn local features with a small receptive field proven by ResNet and VGG, and incrementally increase the number of filters of the same size to extract the high-level neural features from spectrograms. Thus, we employed the number of filters for convolutional layers, which resulted in the best performance on the validation set.

After the last convolution block of VA blocks, we applied channel-wise visual attention and spatial visual attention modules to obtain fine-grained features. We concatenated the VA blocks by adding convolutional features. However, we subsequently performed spatial and channel-wise attention for features captured from convolution blocks, so that features can determine where to focus and what is meaningful on feature maps. These modules assist the model in learning target-specific features of spatial and channel-wise aspects by passing the fine-grained feature from an earlier VA block to a later VA block. Our VA modules were inspired by the CBAM by Woo et al. [47]. They jointly exploit the inter-channel relationship initially investigated by Hu et al. [48] and the inter-spatial relationship initially investigated by Zagoruyko and Komodakis [49]. High recognition performance in object detection research is obtained with CBAM by exploiting the inter-channel relationship of features, and then exploiting the inter-spatial relationship of the channel-wise refined feature. Our VA module is similar to CBAM, but we devised a suitable VA module to refine the high-level neural features. We denoted the height of the feature map as H, and the width of the feature map as W. The channel-wise refined features $F_{c}\in R^{1\;\times \;1\;\times \;C}$ from the channel-wise attention module are represented to emphasize the informative features according to the channel relationship in the learned feature map $V\in R^{H\;\times \;W\;\times \;C}$, as follows:

(1) $$F_{c}=\;\sigma \left(W_{1}\left(W_{0}\left(GAP\left(V\right)\right)\right)+W_{1}\left(W_{0}\left(GMP\left(V\right)\right)\right)\right).$$

Here,  σ is the sigmoid activation. $W_{0}\in R^{1\;\times \;1\;\times \;C/r}$ and $W_{1}\in R^{1\;\times \;1\;\times \;C}$ are parameters with a multi-layer perceptron (MLP) layer, where r is the reduction ratio for the squeeze layer to emphasize the informative feature between channels.

Our channel-wise attention module squeezes and restores the number of filters to aggregate and emphasize inter-channel relationships in feature maps and applies sigmoid activation to parameterize squeezed weight around the non-linearity. In [47,48,50], it is reported that the bottleneck configuration with two FC layers, which reduces and restores the number of filters, is useful to emphasize channel-wise dependencies. Sigmoid functions are inherently non-linear and thus the neural networks, perform channel-wise recalibration by multiplying a value between 0 and 1 for each channel of the feature maps. In addition, we applied global average pooling (GAP) and global max pooling (GMP) to generate channel descriptors that include static information in feature maps. Woo et al. argued that max-pooling and average pooling which are useful for obtaining channel-wise refined features. Max-pooling encodes the degree of the most salient part, whereas average pooling encodes global features to the channel. However, in previous studies [51,52], because GAP aggregates channel-wise statistics of each feature map into one feature map, it forces the feature map to recognize certain elements within the spectral features related target class. Additionally, GAP reduces overfitting because there are no parameters to be learned in the global average pooling layer, reported in [51]. The GMP focuses on a highly localized area on spectral features to find interpretable features within feature maps. The GMP’s advantage with localized features is aligned with previous research [53,54]. Therefore, we argue that it is useful for GAP, which represents the global feature of all channels and GMP, which extracts the most specific portion of all channels to refine deep spectral features in the channel axis in learning SER spectral features.

Next, we utilized our spatial attention module to find the inter-spatial relationship in the feature map. Spatial attention encodes the spatial area of features by aggregating the local feature at spatial position to capture inter-spatial relationship [17]. Spatial attention is commonly constructed by computing statistics across the channel dimension. We obtained a spatially refined feature map $F_{s}\in R^{H\;\times \;W}$ as follows:

(2) $$F_{s}=\;\sigma \left(conv_{1}^{7\;\times \;7}\left[AvgPool\left(V\right);MaxPool\left(V\right)\right]\right).$$

Here, σ is the sigmoid activation. $conv_{1}^{7\;\times \;7}$ denotes a 7 × 7 convolution operation with 1 × 1 stride, aggregating high-level features to a feature map having one channel. AvgPool is the average operation of values across the channel dimension, MaxPool, and is the maximum operation of values across channel dimensions.

For the feature map $V_{\;}$ from the convolution layer, we computed the average and max pooling operations across the channel axis. Then, we concatenated the average and maximum-pooled features and apply a convolution layer. These processes are similar to CBAM, but the convolution layer is different. The CBAM adopts a standard 7 × 7 convolution layer, but we used unbiased 7 × 7  convolution with a 1 × 1 stride and activated refined features using sigmoid. Because the convolution layer calculates spatial attention, we used an unbiased convolution layer to reduce the risk of model overfitting due to bias that diversifies the model computation. In addition, we set the number of channels to one to aggregate and regularize the spatial information on average- and max-pooled features.

The CBAM applies the spatial attention module to a channel-wise refined feature and adopts sigmoid activation, which is argued to show better performance. However, we applied the spatial attention module and channel-wise attention module separately. By separately extracting the spatial and channel relationship information in the feature map extracted from the convolution block, the relationship of the original characteristic can be more widely detected. In addition, we computed the element-wise sum of spatial refined features and channel-refined features to reduce information transformation for learning high-level neural features.

The features from the third VA block with the convolution block and both spatial and channel-wise attention modules were normalized in group normalization and ReLU activation. In addition, we obtained an average pooled feature map with 1 × 1 × 288, calculating the average for feature maps. The average pooled feature map enters a fully connected (FC) layer with 256 neurons, limiting redundancy and the softmax classifier identifies emotions using the log-mel spectrogram.

This model was previously trained with large datasets such as Toronto Emotional Speech Set (TESS) [55] and the Ryerson Audio-Visual Database of Emotional Speech and Song (RAVDESS) for detecting emotions in log-mel spectrograms. The model architecture and pretrained weights are transferred to the learning process of the target datasets. In the next section, we describe the method of leveraging the pretrained model and visual vocabulary with the BOVW when learning other speech datasets.

### 3.2. Fusing Fine-Tuned Model and Attention Weight with Bag of Visual Words

In this section, we describe the fine-tuning method to learn high-level features of small datasets using pretrained layers for a large dataset. Transfer learning shows better performance to freeze the initial several layers and fine-tuned other layers when the target domain dataset is different from the source domain dataset and has fewer training samples than the sourced domain dataset. Speech datasets have a close relationship, including emotions, but they have different features such as language, intensity, and speed. Therefore, when taking the pretrained layers on a large dataset, we freeze layers up to the first VA block and unfreeze and retrain other layers. In the fine-tuning model, we downsampled feature maps to 7 × 7 with 2 × 2 average pooling to aggregate feature maps while preserving the localization of high-level neural features. Furthermore, we reshaped feature maps to 49 × 288 to jointly learn feature vectors from the BOVW and to adapt to the FC layer.

**Figure 3:**
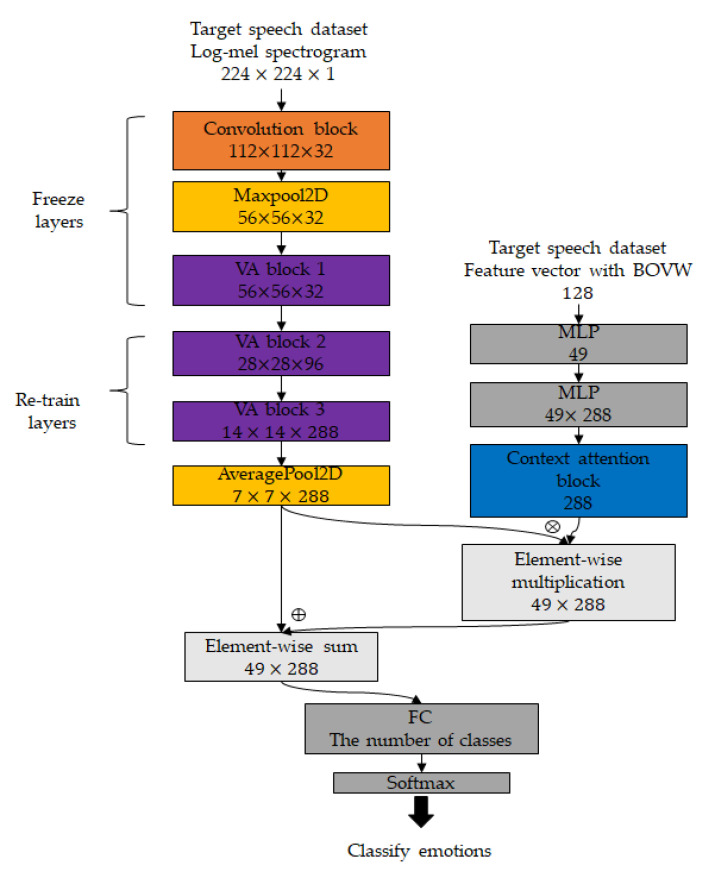
Architecture of the proposed fine-tuned model.

In addition, to learn specific features of the target dataset, we jointly learned the feature vector extracted by analyzing the log-mel spectrogram through the BOVW and fine-tuned model. The proposed fine-tuned model is shown in Figure 3.

We ensemble the BOVW and fine-tuned models to improve classification performance by learning features of both feature vectors expressing which part of the log-mel spectrogram primarily appears according to emotion and high-level neural features learned by the VACNN model. The BOW is a feature extraction technique primarily used in NLP and represents each sentence or document as a feature vector with each word by counting the number of times each word appears in a document. The BOW model is also applicable for vision-recognition research as BOVW. We use the BOVW to represent the features of the log-mel spectrogram in three steps. First, we extracted local features from images using the KAZE descriptor, which is robust to rotation, scale, and limited affine. Then, we divided the feature space through a clustering algorithm as K-means clustering and defined the center point of each group as a visual word. In this step, we used the silhouette value, which measures how similar a point is to its own cluster compared to other clusters. In previous studies [56,57], it is reported that silhouette achieves the best result in most case. In addition, [57] reported that the silhouette shows most robust performance among cluster validity indices in their research. Given observation p, let $ds_{p}$ be the average dissimilarity between the data point and all other points in its own cluster. Let $do_{p}$ be the lowest average dissimilarity between data points and all data points in another cluster. The silhouette $Sil_{p}$ for each data point p is computed as follows:

(3) $$Sil_{p}=\;\frac{do_{p}-ds_{p}}{\max\left(ds_{p},\;do_{p}\right)}.$$

We computed the average for the silhouette value to range from −1 to 1; an average silhouette value close to 1 indicates that the point is correctly placed. We computed the average silhouette value for visual words, and then selected the largest number of clusters that show a high silhouette value.

Finally, we created histograms with 128 visual words as feature vectors to represent the features of the log-mel spectrogram. We employed the number of visual words that resulted in the best performance on the validation set. The feature vector is entered into the MLP layer with 49 neurons to squeeze the information of the feature vector. We expanded the dimensions and entered the MLP layer with 288 neurons to assist the fine-tuned model.

In addition, we applied context attention to assist the fine-tuned model to jointly learn the refined feature vector. Whereas the aforementioned visual attention emphasizes the salient features on the feature map learned by the convolution layer, context attention compresses all the necessary information of sequence data into a fixed-length vector in a different manner with visual attention. In our implementation, the attention vectors are computed and applied to features extracted from the VACNN, as follows:

(4) $${\mathrm{SC}}_{ts}=h_{t}⊺\cdot Wh_{s},$$

(5) $$\alpha_{\mathrm{ts}}=\;\frac{\exp\left(SC_{ts}\right)}{\sum_{i=1}^{n}\exp\left(SC_{i}\right)},$$

(6) $$C_{t}=\;\sum_{s}\alpha_{ts}\cdot h_{s},$$

(7) $$av_{t}=\tanh\left(W_{c}\left[C_{t};h_{t}\right]\right),$$

(8) $$av_{fs}=\tanh\left(Oav_{t}\right)+h_{s}.$$

Here, $\;h_{t}$ denotes the last hidden state of the refined feature vector for the target sequence, denotes the dot product, and $h_{s}$ denotes all hidden states of the refined feature vector for the input sequence. i denotes the index of hidden states, n denotes the number of hidden states, and denotes concatenate. $W_{c}$ is a learnable parameter with an MLP. In addition, O is extracted features using the VACNN model. We utilize all the hidden states of the refined feature vector $h_{s}$ to calculate scores ${\mathrm{SC}}_{ts}$ using Luong’s multiplicative style [58], which generalizes all the hidden states with unbiased MLP and dot product to the last hidden state to compare their similarity. Then, we employed softmax on these scores to produce the attention weights $\alpha_{\mathrm{ts}}$ as a probabilistic interpretation to which state i is to be paid or to be ignored. The context vector $C_{t}$ is generated by performing an element-wise multiplication of $\alpha_{\mathrm{ts}}$ with each state of $h_{s}$. The context vector is generated using the sum of the hidden states weighted by the attention weight. We can attain an attention vector $av_{t}$ by applying a hyperbolic tangent (tanh) for the concatenated context vector $C_{t}$ and the last hidden state $h_{t}$. For joint learning with feature vectors, we fed the attention vector $av_{t}$ into feature maps F of the VACNN using element-wise multiplication, and used tanh as the nonlinear activation. Then, we can attain summarized features $av_{fs}$ by computing the element-wise sum with $h_{s}$ to enforce efficiency of learning salient features in both high-level neural features in the VACNN and the feature vector with the BOVW. We applied ReLU activations $av_{fs}$ and then classified emotions using a classification layer and a softmax classifier with output classes.

## 4. Results and Discussion

### 4.1. Dataset

#### 4.1.1. Berlin Database of Emotional Speech

The EmoDB [20], a benchmark dataset, is a Berlin emotional speech database produced by Burkhardt. The EmoDB covers the following seven emotion categories: anger, boredom, neutral, disgust, fear, happiness, and sadness. The voice was recorded by five male and five female actors between the ages of 20 and 30. The speech corpus consists of 10-sentence German phrases with different lengths, and the total number of utterance files is 535.

#### 4.1.2. Ryerson Audio-Visual Database of Emotional Speech and Song

RAVDESS [19], a benchmark dataset, is a multimodal database used to recognize emotions with facial expression and voice data (speech, song). It primarily includes three categories: voice-only, face-and-voice, and face-only. Speeches were recorded with eight emotions—neutral, calm, happiness, sadness, anger, fear, surprise, and disgust, by 24 professional actors consisting of 12 women and 12 men. Additionally, each speech was recorded separately with strong intensity and normal intensity to express aspects of the voice based on the degree of emotion. We use voice-only data with 1440 utterance files to classify emotions using only utterances.

#### 4.1.3. Toronto Emotional Speech Set

TESS [55], a benchmark dataset, is an English emotional speech database recorded by two female actors recruited from the Toronto area. It has seven emotions: anger, disgust, fear, happiness, pleasant surprise, sadness, and neutral. The female actors, aged 26 and 64 years, spoke a set of 200 target words for each of the seven emotions as well as with the carrier phrase “say the word.” Thus, the speech dataset consisted of a total of 2800 files with 400 sentences recorded for each emotion.

#### 4.1.4. Surrey Audio-Visual Expressed Emotion

SAVEE [21], a benchmark dataset, is a multimodal database used to recognize emotions with facial expressions and audio speech datasets. It is recorded by four male actors, aged between 27 and 31. Speech is recorded as seven emotions related to anger, disgust, fear, happiness, sadness, surprise, and neutral. It has a speech data set of 480 sentences. Because we aim to identify the emotion of the speaker, we use only the speech dataset. Table 1 shows an overview of each speech dataset used.

### 4.2. Experiments

We propose a CNN-based model with spatial and channel-wise visual attention modules to identify emotions in utterances and learn large speech datasets as prework. In addition, we propose a fine-tuning method to improve the performance of classification for speech datasets using a pretrained model and feature vector extracted by the BOVW. To verify the performance of our proposed method, we first selected a large dataset to create a pretrained model. The TESS dataset has the largest number of audio files among the four speech datasets in EmoDB, RAVDESS, SAVEE, and TESS. However, because TESS is recorded by only two speakers, overfitting is a concern in experiments. Therefore, we used both TESS, which has two speakers and RAVDESS, which has 24 speakers for the pretraining dataset. To verify the performance of SER and cross-corpus SER, we tested for EmoDB, SAVEE, and RAVDESS separately.

The log-mel spectrogram has been shown to effectively distinguish features in SER through SER research. When extracting the log-mel spectrogram in utterances, the fast Fourier transformation window length is 2048 and the hop length is 512. For training in our model, the log-mel spectrogram is represented as a 2D grayscale image with 224 × 224 pixels.

For each experiment, we divided the data such that 80% was used for training and 20% for testing. We performed 5-fold cross-validation. In 5-fold cross validation, each fold was iteratively selected to test the model, and the remaining four folds were used to train the model [59,60]. For group normalization, we fix the number of groups to 8. Also, for channel-wise attention in our VA module, we fix the reduction ratio to 4. We use a batch size of 8, and the cross-entropy criterion is used as the training objective function. The Adam algorithm is adopted for optimization. A group normalization layer is adopted in VACNN to avoid overfitting. The model was trained with a 0.001 learning rate and a decay one later every 10 epochs. To avoid overfitting, we used l2 weight regularization with factor 0.0001.

To avoid overfitting, we adopted regularization methods in the experiments. First, early stopping is used to train the model to prevent over-fitting. Overfitting occurs when the learning degree is excessive, and underfitting occurs when the learning degree is poor. Early stopping is a continuously monitored error during training through early stopping. When performance on the validation dataset starts to decrease it stops model training before it overfits the training dataset. Thus, it can reduce overfitting and improve the model generalization performance.

As another method to prevent overfitting, model selection was used in our experiments. Model selection is a method that selects a model from a group of candidate models. Model selection designated the most predictive and fitted model based on the validation accuracy for SER.

#### 4.2.1. Results and Performance of VACNN for Pretraining

We used the VACNN architecture to train the TESS and RAVDESS datasets for pretraining. To train the two datasets simultaneously, the speech data label was set as anger, disgust, fear, happiness, surprise, sadness, and neutral. The batch size is 8 in the whole training process. The cross-entropy criterion was used as the training objective function. The Adam algorithm was adopted for optimization. A group normalization layer is adopted in the VACNN to avoid overfitting. First, we validate the TESS and RAVDESS datasets using the VACNN model for pretraining. To match the label with TESS, the calm label of RAVDESS was recognized as a neutral emotion for pretraining. Table 1 shows the performance, class level precision, recall, F1-score, and overall accuracy tested in TESS and RAVDESS using the VACNN. Precision refers to the percentage of results that are relevant. Recall refers to the percentage of total relevant results correctly classified by the algorithm. The F1-score is a statistical feature defined as the harmonic mean between precision and recall. The overall accuracy is calculated by summing the number of correctly classified values and dividing by the total number of values.

Table 2 enumerates the performance of the proposed VACNN model over the TESS and RAVDESS, which indicates the effectiveness of the model in recognizing emotions with an overall accuracy of 89.03%. In precision, all emotions are recognized with more than 80% class accuracy. Anger showed the highest accuracy of 94%. Fear and surprise showed the highest accuracy of 94%, whereas happiness showed the lowest accuracy of 75%.

#### 4.2.2. Results and Performance of Cross-Corpus SER Test with Fine-Tuned VACNN and BOVW

To test the cross-corpus SER performance, we use the pretrained VACNN with TESS and RAVDESS and apply it to learning the target dataset. When learning the target dataset, we freeze the pretrained weight to the first VA block, retrain layers up to the last average pooling operation in the VACNN architecture, and jointly learn the feature vector extracted by the BOVW through a context attention mechanism. We tested cross-corpus SER performance for SAVEE, RAVDESS, and EmoDB. For fine-tuning process, we recognized emotions for RAVDESS with eight emotions. Table 3 presents the overall accuracy performance of the VACNN + BOVW with and without fine-tuning. In addition, Table 3 shows that the overall accuracy performance of the model has the same structure as the fine-tuned VACNN structure, but the CBAM module is applied instead of our VA module to compare the performance of the VA and CBAM modules.

For each dataset, the transferred VACNN + BOVW with a pretrained layer on the other dataset showed higher accuracy than the purely trained VACNN + BOVW. In addition, our fine-tuned model shows a higher performance than the VACNN using CBAM instead of the VA module.

Table 4, Table 5 and Table 6 represent the SER performance in terms of precision, recall, and F1-score for the tested RAVDESS, EmoDB, and SAVEE datasets, respectively, for pretraining with TESS and RAVDESS.

Table 4 shows the precision, recall, and F1-score for eight emotions for RAVDESS. Our method shows an overall accuracy of 83.33% for RAVDESS. In terms of precision, calm emotion showed 95% highest accuracy in the eight emotions, whereas sadness and neutral emotion showed 74% accuracy. In recall, anger showed the highest accuracy of 91%, whereas happiness showed the lowest accuracy of 74%.

Table 5 shows the precision, recall, and F1-score for seven emotions for EmoDB. Our method shows an overall accuracy of 86.92% for EmoDB. In precision, disgust registered at 95% and had the highest accuracy, whereas fear showed the lowest accuracy of 74%. In recall, sadness showed the highest accuracy of 96%, whereas happiness showed the lowest accuracy of 73%.

Table 6 shows the precision, recall, and F1-score for seven emotions for SAVEE. Our method shows an overall accuracy of 75% for SAVEE. In terms of precision, neutral emotion achieved 88% and had the highest accuracy in the seven emotions, whereas surprise showed the lowest accuracy of 59%. In recall, neutral emotions showed the highest accuracy of 93%, while anger showed the lowest accuracy of 58%.

Table 7, Table 8 and Table 9 show the confusion matrix in RAVDESS, EmoDB, and SAVEE, respectively, for the cross-corpus SER test.

In Table 7, RAVDESS shows the highest performance in anger, and anger is confused with disgust, happiness, and surprise. In addition, sadness shows the lowest accuracy and is confused with neutral emotions.

In Table 8, EmoDB shows the highest accuracy with disgust, and disgust is confused with fear. In addition, happiness shows the lowest accuracy and is confused with neutral and disgust.

In Table 9, SAVEE showed the highest accuracy in neutral, and neutral was confused with sadness. Anger showed the lowest accuracy and confused surprised and happiness.

As interest in cross-corpus SER increased, we proposed pretraining on a large dataset with TESS and RAVDESS and a fine-tuned model when learning a relatively small dataset with RAVDESS, EmoDB, and SAVEE. Table 10 compares the performance with cross-corpus SER studies in SAVEE, EmoDB, and RAVDESS, respectively. Latif et al. used latent codes extracted by 88 low-level features related to spectral energy, frequency, cepstral features, and dynamic information based on a generative adversarial network (GAN). They pretrained the source dataset with SVM and transferred the feature space to the target dataset. Mao et al. [61] pretrained the local invariant feature extracted by a sparse autoencoder on the raw spectrogram with SVM for the source dataset and transferred feature space with SVM to identify emotions on the target dataset. Goel and Beigi [62] extracted 384 features related to ZCR, root-mean-square energy, pitch, and pretrained source dataset with LSTM and a fine-tuned model to learn the low-level features of the target dataset. Mustaqeem and Kwon designed a stride deep CNN model and learned the high-level features on a raw spectrogram and tested it in the target dataset. It was shown that there have been many studies on cross-corpus SER performance, mainly transferring the feature space or fine-tuned high-level features. Milner et al. [63] extracted log-mel filterbanks with 23 dimensions and a pretrained source dataset with BiLSTM and transferred the model to learn the target dataset. Table 10 shows that our fine-tuned method outperforms the existing method in RAVDESS, EmoDB, and SAVEE. This shows that it is more efficient to jointly retrain with the BOVW feature vectors and fine-tuned model for the pretrained layer on a large dataset. Parry et al. [64] extracted MFCC, and pretrained source dataset using LSTM, CNN, and CNN-LSTM. And then they transferred trained weight to the target dataset. To extract features from the target dataset, the proposed method does not simply apply the weights learned from the source dataset with enough training samples, but retrains the weights trained by source dataset to represent discriminative features. Additionally, our method jointly learned local and global features in the log-mel spectrogram through BOVW, so our method considers the global features from the source dataset, and the local features of the target dataset. Thus, it shows better performance than existing cross-corpus SER studies.

In the state-of-the-art methods, researchers have used traditional and un-normalized features for SER. Therefore, we tested and compared demonstrated other baseline CNN models and VACNN for cross-corpus SER. In order to evaluate CNN base models as a feature extractor. We pretrained TESS and RAVDESS, and tested RAVDESS, EmoDB, and SAVEE. The comparative analysis of other baseline CNN model and VACNN is given in Table 10. VACNN showed better performance than ResNet, Inception v3 [65], and MobileNet [66].

## 5. Conclusions

With the development of ER technology, research on SER is emerging. The SER technology primarily classifies emotions by learning a set of low-level features or spectral features.

One method classifies emotions based on feature space for low-level features, including pitch and energy, using SVM and GMM. Another method uses spectral features, including a log-mel spectrogram, STFT, to learn high-level spectral features to identify emotions using CNN and LSTM.

Currently, various speech datasets, such as SAVEE, EmoDB, and RAVDESS, are pronounced by speakers of different languages and ages. Therefore, SER studies focus on the cross-corpus SER problem to recognize emotions in different speech datasets. Accordingly, recent research has tested experiments that train ER models in speech datasets and test for learning of other speech datasets but exhibited an ER accuracy performance of 50% to 60%.

Transfer learning is a machine-learning technique that shows performance improvement when fine-tuned models use pretrained weights on other related datasets. We pretrain the log-mel spectrogram on both TESS and RAVDESS using our proposed VACNN model and learn other datasets using a fine-tuned model with BOVW features. The VACNN model applies a visual attention module for channel-wise and spatial attention. To pretrain the TESS and RAVDESS, we design the VACNN model, which refines feature maps to emphasize the important parts of the high-level spectral features through channel-wise and spatial attention modules to the next block. To learn the target dataset, the BOVW is used to represent the feature vector of the log-mel spectrogram. The feature vector is expressed as the attention weight to support the fine-tuned VACNN model on the log-mel spectrogram of the target dataset. The fine-tuned VACNN considers the generic features of the pretrained layer and the target-specific features of the retraining layer. In addition, a fine-tuned VACNN jointly learn the local and global features extracted by BOVW. Thus, our method exhibits better performance than existing cross-corpus SER studies that consider the discrepancy of feature distribution of the source and target dataset. Our proposed method was tested for performance in RAVDESS, EmoDB, and SAVEE, and shows improved overall accuracy compared to the methods used to existing cross-corpus SER methods. However, because we consider only the log-mel spectrogram as input data, our method ignores low-level features related to emotions. Therefore, future work may include low-level features for cross-corpora to improve the results of SER by classifying them with spectral and low-level features.

## Figures and Tables

**Table 1:** Overview of the selected datasets.

Dataset	Language	Utterances	Emotions	Emotion Labels
EmoDB	German	565	7	Anger, Boredom, Neutral, Disgust, Fear, Happiness, Sadness
RAVDESS	English	1440	8	Neutral, Calm, Happiness, Sadness, Anger, Fear, Surprise, Disgust
TESS	English	2800	7	Anger, Disgust, Fear, Happiness, Pleasant Surprise, Sadness, Neutral
SAVEE	English	480	7	Anger, Disgust, Fear, Happiness, Sadness, Surprise, Neutral

**Table 2:** Confusion matrix based on TESS and RAVDESS dataset for pretraining.

Emo Class	Precision	Recall	F1-Score
Anger	0.94	0.88	0.91
Disgust	0.89	0.92	0.90
Fear	0.83	0.94	0.88
Happiness	0.90	0.75	0.82
Neutral	0.92	0.89	0.91
Sadness	0.83	0.90	0.87
Surprise	0.93	0.94	0.93
Average	0.89	0.89	0.89

**Table 3:** Comparison of overall accuracy using the proposed VACNN + BOVW, fine-tuned VACNN + BOVW, and fine-tuned VACNN with CBAM instead VA module + BOVW.

Dataset	Pure VACNN + BOVW	Fine-Tuned VACNN + BOVW	Fine-Tuned VACNN (CBAM) + BOVW
RAVDESS	0.72	0.83	0.77
EmoDB	0.77	0.87	0.81
SAVEE	0.69	0.75	0.72

**Table 4:** Cross-corpus emotion recognition result using proposed fine-tuning method on RAVDESS.

Emo Class	Precision	Recall	F1-Score
Anger	0.77	0.91	0.83
Calm	0.95	0.89	0.92
Disgust	0.89	0.85	0.87
Fear	0.91	0.84	0.87
Happiness	0.81	0.74	0.77
Neutral	0.74	0.88	0.80
Sadness	0.74	0.76	0.75
Surprise	0.86	0.86	0.85
Average	0.83	0.83	0.83

**Table 5:** Cross-corpus emotion recognition result using proposed fine-tuning method on EmoDB.

Emo Class	Precision	Recall	F1-Score
Anger	0.94	0.91	0.93
Boredom	0.87	0.87	0.88
Disgust	0.95	0.95	0.97
Fear	0.74	0.80	0.77
Happiness	0.86	0.73	0.79
Neutral	0.80	0.85	0.81
Sadness	0.86	0.96	0.91
Average	0.86	0.87	0.87

**Table 6:** Cross-corpus emotion recognition result using proposed fine-tuning method on SAVEE.

Emo Class	Precision	Recall	F1-Score
Anger	0.74	0.58	0.65
Disgust	0.81	0.65	0.72
Fear	0.59	0.76	0.67
Happiness	0.72	0.59	0.65
Neutral	0.88	0.93	0.90
Sadness	0.73	0.83	0.78
Surprise	0.59	0.65	0.67
Average	0.72	0.71	0.72

**Table 7:** Confusion matrix for emotion prediction on RAVDESS using RAVDESS and TESS.

Emo Class	Anger	Calm	Disgust	Fear	Happiness	Neutral	Sadness	Surprised
Anger	0.91	0.00	0.03	0.00	0.03	0.00	0.00	0.03
Calm	0.00	0.89	0.00	0.00	0.05	0.02	0.05	0.00
Disgust	0.10	0.02	0.85	0.00	0.00	0.00	0.03	0.00
Fear	0.00	0.00	0.03	0.84	0.03	0.00	0.08	0.02
Happiness	0.07	0.00	0.02	0.04	0.74	0.02	0.04	0.07
Neutral	0.00	0.00	0.00	0.00	0.12	0.88	0.00	0.00
Sadness	0.05	0.03	0.03	0.03	0.03	0.08	0.76	0.00
Surprise	0.00	0.00	0.00	0.06	0.02	0.00	0.06	0.86

**Table 8:** Confusion matrix for emotion prediction on EmoDB using RAVDESS and TESS.

Emo Class	Anger	Boredom	Disgust	Fear	Happiness	Neutral	Sadness
Anger	0.91	0.00	0.00	0.04	0.05	0.00	0.00
Boredom	0.00	0.87	0.00	0.00	0.00	0.10	0.03
Disgust	0.00	0.00	0.95	0.05	0.00	0.00	0.00
Fear	0.04	0.00	0.00	0.8	0.00	0.04	0.12
Happiness	0.00	0.00	0.04	0.15	0.73	0.08	0.00
Neutral	0.03	0.12	0.00	0.00	0.00	0.85	0.00
Sadness	0.00	0.00	0.00	0.00	0.00	0.04	0.96

**Table 9:** Confusion matrix for emotion prediction on SAVEE using RAVDESS and TESS.

Emo Class	Anger	Disgust	Fear	Happiness	Neutral	Sadness	Surprised
Anger	0.58	0.08	0.08	0.13	0.00	0.00	0.13
Disgust	0.00	0.65	0.07	0.00	0.12	0.08	0.08
Fear	0.00	0.05	0.76	0.05	0.05	0.05	0.05
Happiness	0.22	0.00	0.05	0.59	0.00	0.00	0.14
Neutral	0.00	0.00	0.00	0.00	0.93	0.07	0.00
Sadness	0.00	0.04	0.00	0.00	0.13	0.83	0.00
Surprise	0.00	0.3	0.05	0.00	0.00	0.00	0.65

**Table 10:** Comparison of our method and related works on RAVDESS, EmoDB, SAVEE for cross-corpus SER.

Dataset	Method	Features	Overall Accuracy
RAVDESS	Mustaqeem and Kwon [33]	Raw spectrogram	56.50
	Milner et al. [63]	Log-mel spectrogram	75.60
	Parry et al. [64]	MFCC	65.67
	Inception v3 [65]	Log-mel spectrogram	69.10
	MobileNet [66]	Log-mel spectrogram	71.53
	ResNet [46]	Log-mel spectrogram	73.26
	VACNN	Log-mel spectrogram	74.31
	VACNN + BOVW	Log-mel spectrogram	83.33
EmoDB	Zong et al. [36]	Low-level acoustic features	61.41
	Latif et al. [41]	Low-level acoustic features + latent code	65.30
	Mao et al. [61]	Raw spectrogram	71.80
	Parry et al. [64]	MFCC	69.72
	Inception v3 [65]	Log-mel spectrogram	73.83
	MobileNet [66]	Log-mel spectrogram	76.64
	ResNet [46]	Log-mel spectrogram	76.64
	VACNN	Log-mel spectrogram	79.44
	VACNN + BOVW	Log-mel spectrogram	86.92
SAVEE	Latif et al. [41]	Low-level acoustic features + latent code	53.20
	Goel and Beigi [62]	Low-level acoustic features	55.55
	Mao et al. [61]	Raw spectrogram	57.2
	Parry et al. [64]	MFCC	72.66
	Inception v3 [65]	Log-mel spectrogram	51.04
	MobilNet [66]	Log-mel spectrogram	56.25
	ResNet [46]	Log-mel spectrogram	59.38
	VACNN	Log-mel spectrogram	64.58
	VACNN + BOVW	Log-mel spectrogram	75.00

## Abbreviation

SER	Speech Emotion Recognition
VACNN	Visual Attention Convolutional Neural Network
TESS	Toronto Emotional Speech Set
RAVDESS	Ryerson Audio-Visual Database of Emotional Speech and Song
EmoDB	Berlin Database of Emotional Speech
SAVEE	Surrey Audio-Visual Expressed Emotion
ER	Emotion Recognition
HCI	Human-Computer Interaction
MFCC	Mel-Frequency Cepstral Coefficient
TEO	Teager Energy Operator
HMM	Hidden Markov Model
GMM	Gaussian Mixture Model
SVM	Support Vector Machine
RNN	Recurrent Neural Network
DNN	Deep Neural Network
CNN	Convolutional Neural Network
LSTM	Long Short-Term Memory
STFT	Short-Time Fourier Transform
BOVW	Bag of Visual Words
SIFT	Scale-Invariant Feature Transform
HOG	Histogram of Oriented Gradients
SURF	Speeded-Up Robust Features
NLP	Natural Language Processing
KNN	K-Nearest Neighbor
ZCR	Zero-Crossing Rate
DBN	Deep Belief Network
RBM	Restricted Boltzmann Machine
IEMOCAP	Interactive Emotional Dyadic Motion Capture
GN	Group Normalization
ReLu	Rectified Linear Unit
ResNet	Residual Neural Network
CBAM	Convolutional Block Attention Module
SEnet	Squeeze and Excitation Network
GAP	Global Average Pooling
GMP	Global Max Pooling
FC	Fully Connected
BOW	Bag of Words
MLP	Multi-Layer Perceptron
Tanh	Hyperbolic Tanh
GAN	Generative Adversarial Network
Bi-LSTM	Bidirectional Lstm
FFT	Fast Fourier Transformation
CASIA	Chinese Emotional Speech Corpus
RDA	Regularized Discriminant Analysis
SFFS	Sequential floating forward selection
RML	Ryerson Multimedia Laboratory
A-LSTM	Advanced Long Short-term Memory
ABC	Airplane Behavior Corpus

## References

[1] Bachmann D., Weichert F., Rinkenauer G. (2018). Review of Three-Dimensional Human-Computer Interaction with Focus on the Leap Motion Controller. Sensors.

[2] Akcay M.B., Oguz K. (2019). Speech emotion recognition: Emotional models, databases, features, preprocessing methods, supporting modalities, and classifiers. Speech Commun..

[3] Rajan S., Chenniappan P., Devaraj S., Madian N. (2019). Facial expression recognition techniques: A comprehensive survey. IET Image Process..

[4] Li T.M., Chao H.C., Zhang J. (2019). Emotion classification based on brain wave: A survey. Hum. Cent. Comput. Inf. Sci..

[5] Minaee S., Abdolrashidi A., Su H., Bennamoun M., Zhang D. (2019). Biometric Recognition Using Deep Learning: A survey. arXiv.

[6] Luengo I., Navas E., Hernandez I. (2010). Feature analysis and evaluation for automatic emotion identification in speech. IEEE Trans. Multimed..

[7] Ozsever T. (2019). A novel feature selection method for speech emotion recognition. Appl. Acoust..

[8] Zhu L., Chen L., Zhao D., Zhou J., Zhang W. (2017). Emotion Recognition from Chinese Speech for Smart Affective Services Using a Combination of SVM and DBN. Sensors.

[9] Sun L., Zou B., Fu S., Chen J., Wang F. (2019). Speech emotion recognition based on DNN-decision tree SVM model. Speech Commun..

[10] Hamid L.A. (2020). Egyptian Arabic speech emotion recognition using prosodic, spectral and wavelet features. Speech Commun..

[11] Zhang S., Zhang S., Huang T., Gao W. (2018). Speech Emotion Recognition Using Deep Convolutional Neural Network and Discriminant Temporal Pyramid Matching. IEEE Trans. Multimed..

[12] Chen M., He X., Yang J., Zhang H. (2018). 3-D Convolutional Recurrent Neural Networks with Attention Model for Speech Emotion Recognition. IEEE Signal Process. Lett..

[13] Xie Y., Liang R., Liang Z., Huang C., Zou C., Schuller B. (2019). Speech Emotion Classification Using Attention-Based LSTM. IEEE ACM Trans. Audio Speech Lang. Process..

[14] Sun L., Chen J., Xie K., Gu T. (2018). Deep and shallow features fusion based on deep convolutional neural network for speech emotion recognition. Int. J. Speech Technol..

[15] Huang C., Narayanan S. Attention Assisted Discovery of Sub-Utterance Structure in Speech Emotion Recognition. Proceedings of the Interspeech 2016.

[16] Tan C., Sun F., Kong T., Zhang W., Yang C., Liu C. A Survey on Deep Transfer Learning. Proceedings of the 27th International Conference on Artificial Neural Networks.

[17] Chen L., Zhang H., Xiao J., Nie L., Shao J., Liu W., Chua T.S. SCA-CNN: Spatial and Channel-wise Attention in Convolutional Networks for Image Captioning. Proceedings of the IEEE Conference on Computer Vision and Pattern Recognition.

[18] Wang R., Ding K., Yang J., Xue L. (2016). A novel method for image classification based on bag of visual words. J. Vis. Commun. Image Represent..

[19] Livingstone S.R., Russo F.A. (2018). The Ryerson Audio-Visual Database of Emotional Speech and Song (RAVDESS): A dynamic, multimodal set of facial and vocal expressions in North American English. PLoS ONE.

[20] Burkhardt F., Paeschke A., Rolfes M., Sendlmeier W., Weiss B. A database of german emotional speech. Proceedings of the Interspeech.

[21] Jackson P., Haq S. (2014). Surrey Audio-Visual Expressed Emotion (SAVEE) Database.

[22] Institute of Automation, Chinese Academy of Sciences (2005). CAISA Mandarin Emotional Speech Corpus. http://www.chineseldc.org/resource_info.php?rid=76.

[23] Kuchibhotla S., Vankayalapati H., Anne K.R. (2016). An optimal two stage feature selection for speech emotion recognition using acoustic features. Int. J. Speech Technol..

[24] Noroozi F., Marjanovic M., Njegus A., Escalera S., Anbarjafari G. (2017). Audio-visual emotion recognition in video clips. IEEE Trans. Affect. Comput..

[25] Wang Y., Guan L. (2008). Recognizing human emotional state from audiovisual signals. IEEE Trans. Multimed..

[26] Martin O., Kotsia I., Macq B., Pitas I. The eNTERFACE’05 audio-visual emotion database. Proceedings of the 22nd International Conference on Data Engineering Workshops (ICDEW’06).

[27] Fahad M., Yadav J., Pradhan G., Deepak A. (2018). DNN-HMM based Speaker Adaptive Emotion Recognition using Proposed Epoch and MFCC Features. arXiv.

[28] Busso C., Bulut M., Lee C.C., Kazemzadeh A., Mower E., Kim S., Chang J.N., Lee S., Narayanan S.S. (2008). IEMOCAP: Interactive emotional dyadic motion capture database. Lang. Resour. Eval..

[29] Misramadi S., Barsoum E., Zhang C. Automatic speech emotion recognition using recurrent neural networks with local attention. Proceedings of the IEEE International Conference on Acoustic, Speech, and Signal Processing.

[30] Liu G., Tao F. Advanced LSTM: A study about better time dependency modeling in emotion recognition. Proceedings of the IEEE International Conference on Acoustic, Speech, and Signal Processing.

[31] Tarantino L., Garner P.N., Lazaridis A. Self-Attention for Speech Emotion Recognition. Proceedings of the Interspeech.

[32] Hajarolasvadi N., Demirel H. (2019). 3D CNN-Based Speech Emotion Recognition Using K-Means Clustering and Spectrograms. Entropy.

[33] Mustaqeem, Kwon S. (2020). A CNN-Assisted Enhanced Audio Signal Processing for Speech Emotion Recognition. Sensors.

[34] Alkaya A., Eker I. (2011). Variance sensitive adaptive threshold-based PCA method for fault detection with experimental application. ISA Trans..

[35] Schuller B., Vlasenko B., Wollmer M., Stuhlsatz A., Wendemuth A., Rigoll G. (2010). Cross-Corpus Acoustic Emotion Recognition: Variances and Strategies. IEEE Trans. Affect. Comput..

[36] Zong Y., Zheng W., Zhang T., Huang X. (2016). Cross-corpus speech emotion recognition based on domain-adaptive least-squares regressions. IEEE Signal Process..

[37] Huang W., Xue W., Mao Q., Zhan Y. (2017). Unsupervised domain adaption for speech emotion recognition using PCANET. Multimed. Tools Appl..

[38] Schuller B., Wimmer M., Arsic D., Rigoll G., Radig B. Audiovisual Behavior Modeling by Combined Feature Spaces. Proceedings of the International Conference on Acoustics, Speech, and Signal Processing.

[39] Zhang W., Song P. (2020). Transfer Sparse Discriminant Subspace Learning for Cross-Corpus Speech Emotion Recognition. IEEE ACM Trans. Audio Speech Lang. Process..

[40] Zhalehpour S., Onder O., Akhtar Z., Erdem C.E. (2016). BAUM-1: A spontaneous Audio-Visual Face Database of Affective and mental States. IEEE. Trans. Affect. Comput..

[41] Latif S., Qadir J., Bilal M. Unsupervised Adversarial Domain Adaptation for Cross-Lingual Speech Emotion Recognition. Proceedings of the Affective Computing and Intelligent Interaction 2019.

[42] Batilner A., Steidl S., Noeth E. Releasing a thoroughly annotated and processed spontaneous emotional database: The FAU Aibo Emotion Corpus. Proceedings of the Satellite Workshop of LREC 2008 on Corpora for Research on Emotion and Affect.

[43] Costantini G., Laderola L., Paoloni A., Todisco M. EMOVO Corpus: An Italian Emotional Speech Database. Proceedings of the Ninth International Conference on Language Resources and Evaluation.

[44] Liu J., Zheng W., Zong Y., Lu C., Tang C. (2020). Cross-Corpus Speech Emotion Recognition Based on Deep Domain-Adaptive Convolutional Neural Network. IEICE Trans. Inf. Syst..

[45] Wu Y., He K. Group Normalization. Proceedings of the European Conference on Computer Vision.

[46] He K., Zhang X., Ren S., Sun J. Deep residual learning for image recognition. Proceedings of the IEEE Conference on Computer Vision and Pattern Recognition.

[47] Woo S., Park J., Lee J., Kwon I.S. CBAM: Convolutional Block Attention Module. Proceedings of the European Conference on Computer Vision.

[48] Hu J., Shen L., Sun G. Squeeze-and-excitation networks. Proceedings of the European Conference on Computer Vision.

[49] Zagoruyko S., Komodakis N. Paying more attention to attention: Improving the performance of convolutional neural networks via attention transfer. Proceedings of the 5th International Conference on Learning Representations.

[50] Zhao P., Zhang J., Fang W., Deng S. (2020). SCAU-Net: Spatial-Channel Attention U-Net for Gland Segmentation. Front. Bioeng. Biotechol..

[51] Lin M., Chen Q., Yan S. (2013). Network in Network. arXiv.

[52] Springenberg J.T., Dosovitskiy A., Brox T., Riedmiller M. (2014). Striving for simplicity: The all convolutional net. arXiv.

[53] Yan Z., Liu W., Wen S., Yang Y. (2019). Multi-label image classification by feature attention network. IEEE Access.

[54] Zhou B., Khosla A., Lapedriza A., Oliva A., Torralba A. Learning deep features for discriminative localization. Proceedings of the Computer Vision and Pattern Recognition.

[55] Pichora-Fuller M.K., Dupuis K. Toronto Emotional Speech Set (TESS). https://tspace.library.utoronto.ca/handle/1807/24487(2010).

[56] Jauhiainen S., Karkkanen T. A simple Cluster Validation Index with Maximal Coverage. Proceedings of the European Symposium on Artificial Neural Networks, Computational Intelligence and Machine Learning.

[57] Vendramin L., Campello R.J.G.B., Hruschka E.R. (2010). Relative clustering validity criteria: A comparative overview. Stat. Anal. Data. Min..

[58] Luong M.T., Pham H., Manning C.D. Effective Approaches to Attention-based Neural Machine Translation. Proceedings of the Empirical Methods on Natural Language Processing 2015.

[59] Chui K.T., Fung D.C.L., Lytras M.D., Lam T.M. (2020). Predicting at-risk university students in a virtual learning environment via a machine learning algorithm. Comput. Hum. Behav..

[60] Liu Z., Dong W., Jiang W., Zili H. (2019). csDMA: An improved bioinformatics tool for identifying DNA 6 mA modifications via Chou’s 5-step rule. Sci. Rep..

[61] Mao Q., Dong M., Huang Z., Zhan Y. (2014). Learning Salient Features for Speech Emotion Recognition Using Convolutional Neural Networks. IEEE Trans. Multimed..

[62] Goel S., Beigi H. Cross-Lingual Cross-Corpus Speech Emotion Recognition. Proceedings of the New York Academy of Science Machine Learning Symposium.

[63] Milner R., Jalal M.A., Ng R.W.M., Hain T. A Cross-Corpus Study Speech Emotion Recognition. Proceedings of the 2019 IEEE Automatic Speech Recognition and Understanding Workshop (ASRU).

[64] Parry J., Palaz D., Clarke G., Lecomte P., Mead R., Berger M., Hofer G. Analysis of Deep Learning Architectures for Cross-corpus Speech Emotion Recognition. Proceedings of the Interspeech.

[65] Szegedy C., Vanhoucke V., Ioffe S., Shlens J., Wojna Z. Rethinking the inception architecture for computer vision. Proceedings of the IEEE Conference on Computer Vision and Pattern Recognition.

[66] Howard A.G., Zhu M., Chen B., Kalenichenko D., Wang W., Weyand T., Andreetto M., Adam H. (2017). Mobilenets: Efficient Convolutional Neural Networks for Mobile Vision Applications. arXiv.

